# Developing transgenic wheat to encounter rusts and powdery mildew by overexpressing barley *chi26* gene for fungal resistance

**DOI:** 10.1186/s13007-017-0191-5

**Published:** 2017-05-22

**Authors:** Hala F. Eissa, Sameh E. Hassanien, Ahmed M. Ramadan, Moustafa M. El-Shamy, Osama M. Saleh, Ahmed M. Shokry, Mohamed Abdelsattar, Yasser B. Morsy, Maher A. El-Maghraby, Hussien F. Alameldin, Sabah M. Hassan, Gamal H. Osman, Hesham T. Mahfouz, Gharib A. Gad El-Karim, Magdy A. Madkour, Ahmed Bahieldin

**Affiliations:** 1 Agricultural Genetic Engineering Research Institute (AGERI), Agriculture Research Center (ARC), Giza, 12619 Egypt; 2 Faculty of Biotechnology, Misr University for Science and Technology (MUST), Post Box 77, 6th October City, Egypt; 3 Department of Biological Sciences, Faculty of Science, King Abdulaziz University, P.O. Box 80141, Jeddah, 21589 Saudi Arabia; 4 Plant Pathology Research Institute (PPRI), Agriculture Research Center (ARC), Giza, 12619 Egypt; 5 National Centre for Radiation Research and Technology (NCRRT), Cairo, 11781 Egypt; 6 Department of Biotechnology, Faculty of Applied Medical Science, Taif University, Turrabah, 21995 Saudi Arabia; 7 Field Crops Research Institute, Agriculture Research Center (ARC), Giza, 12619 Egypt; 8 Plant Soil and Microbial Sciences Department, Michigan State University, East Lansing, MI 48824 USA; 9 Department of Genetics, Faculty of Agriculture, Ain Shams University, Cairo, 11566 Egypt; 10 Department of Biology, Faculty of Applied Sciences, Umm Al-Qura University, Makkah, 21955 Saudi Arabia; 11 Department of Pomology, The Horticulture Research Institute (HRI), Agriculture Research Center (ARC), Giza, 12619 Egypt; 12 Arid Lands Agricultural Research Institute (ALARI), Faculty of Agriculture, Ain Shams University, P.O. Box 68, Hadayek Shoubra, Cairo, 11241 Egypt

**Keywords:** Transgenesis, Southern, qPCR, Chitinase activity, qRT-PCR, Substantial equivalence

## Abstract

**Background:**

The main aim of this study was to improve fungal resistance in bread wheat via transgenesis. Transgenic wheat plants harboring barley *chitinase* (*chi26*) gene, driven by maize *ubi* promoter, were obtained using biolistic bombardment, whereas the herbicide resistance gene, *bar,* driven by the *CaMV 35S* promoter was used as a selectable marker.

**Results:**

Molecular analysis confirmed the integration, copy number, and the level of expression of the *chi26* gene in four independent transgenic events. Chitinase enzyme activity was detected using a standard enzymatic assay. The expression levels of *chi26* gene in the different transgenic lines, compared to their respective controls, were determined using qRT-PCR. The transgene was silenced in some transgenic families across generations. Gene silencing in the present study seemed to be random and irreversible. The homozygous transgenic plants of T4, T5, T6, T8, and T9 generations were tested in the field for five growing seasons to evaluate their resistance against rusts and powdery mildew. The results indicated high chitinase activity at T0 and high transgene expression levels in few transgenic families. This resulted in high resistance against wheat rusts and powdery mildew under field conditions. It was indicated by proximate and chemical analyses that one of the transgenic families and the non-transgenic line were substantially equivalent.

**Conclusion:**

Transgenic wheat with barley *chi26* was found to be resistant even after five generations under artificial fungal infection conditions. One transgenic line was proved to be substantially equivalent as compared to the non-transgenic control.

**Electronic supplementary material:**

The online version of this article (doi:10.1186/s13007-017-0191-5) contains supplementary material, which is available to authorized users.

## Background

Wheat, rice, and maize are the most important food crops, as they contribute to 60% of the food consumed in the world [1]. Food security depends upon our ability to increase the production of cereals concomitant with the growing population [2]. Rust diseases, including leaf rust (*Puccinia recondite Eriks*), yellow or stripe rust (*Puccinia striiformis* Westend), and stem rust (*Puccinia graminis* Pers.: Pers) are the most important foliar diseases in wheat [3], causing yield losses of up to 20% [4]. Plants respond to fungal infection by complex mechanisms. The production of pathogenesis-related (PR) proteins [5–7], such as chitinase and β-1,3-glucanase, is one of the most effective strategies involved in plant immune response [8]. Chitinase (poly[1,4-*N*-acetyl-β-d-glucosamine] glycan hydrolase, EC 3.2.1.14) catalyzes the hydrolysis of β1-4-linkages of *N*-acetylglucosamine of chitin resulting in *N*-acetyl glucosamine oligomers [9]. Chitin is the major component of fungal cell walls [9]; therefore, the ability of chitinase to hydrolyze fungal cell wall can be employed to inhibit the growth of fungal hyphal tips [10, 11]. Resistant wheat cultivars can be obtained via genetic transformation, and deployed to reduce yield losses and protect seed quality deteriorated by rust and powdery mildew diseases. Molecular breeding and transgenesis are two main approaches of developing disease resistant crops [12]. The expression of *chitinase* gene has been found to enhance the resistance against fungal diseases in many plant species via genetic transformation, for instance, the expression of a class II chitinase in *Brassica juncea* could successfully provide protection against *Alternaria* leaf spot disease [13]. A high chitinase activity along with improved β-1,3-glucanase activity in transgenic grapevines enhanced the resistance against downy mildew [14]. The introduction of rice *chitinase* (*RC7*) in Indica rice bestowed considerable resistance against sheath blight [15]. The *AFP*-*CHI* transgenic oriental melons could resist the infection of *Rhizoctonia solani* and *Fusarium oxysporum* [16]. Earlier studies in wheat indicated that the constitutive expression of class II barley chitinase could enhance resistance against *Erysiphe graminis* [17] and *Fusarium graminearum* [18, 19]. The aim of the present work was to evaluate transgenic wheat lines, harboring the barley *chi26* gene for resistance against rust and powdery mildew diseases. The T4, T5, T6, T8, and T9 generations of the four transgenic lines were assayed using artificial infection in the field over five growing seasons. The most promising transgenic family was analyzed in contrast to the non-transgenic controls to substantiate the resistance.

## Methods

### Genetic transformation

**Fig. 1:**
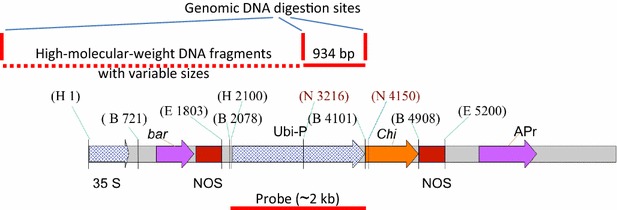
Restriction map of the plant expression vector pBarley/chi/bar. H, *Hin*dIII; B, *Bam*HI; E, *Eco*RI; N, *Nco*I. Sites of probe and genomic DNA digestions are indicated by *red solid* and/or *dotted lines*

The plasmid pBarley/chi/bar (Fig. 1) harboring the full-length barley *chi26* and *bar* genes [20] was used to transform immature embryos of bread wheat (*Triticum aestivum* L.) cv. Hi-Line. The tissue culture and transformation were carried out as reported previously by Sivamani et al. [21]. The obtained primary transformants were transferred to the biocontainment facility at Agricultural Genetic Engineering Research Institute (AGERI), Agriculture Research Center (ARC), Giza, Ministry of Agriculture, Egypt, and assayed using leaf painting with 1 g/L of Basta™ (Bayer Crop Science PVT Ltd). Biosafety measures and guidelines were followed across the nine growing and testing seasons of the transgenic lines and their families.

### Polymerase chain reaction (PCR)

Genomic DNA was extracted from putative transgenic plants, which were resistant to the herbicide Basta™, and the non-transgenic (cv. Hi-Line) control, using the DNeasy™ Plant Mini kit (Qiagen, cat no. 69104). Using PCR, *chi26* (800 bp) as well as *bar* (485 bp) gene fragments were amplified using gene-specific primers (Additional file 1: Table S1). The reaction conditions were optimized in a mixture (50 μL total volume) composed of dNTPs (0.2 mM), MgCl_2_ (1.5 mM), 1× buffer, primers (0.2 μM), template DNA (100 ng), and *Taq* DNA polymerase (2 units). The amplification was carried out in a Hybaid PCR Express system. The system was programmed for 40 cycles as follows: 94 °C for 4 min (1 cycle); 94 °C for 1 min, 58 °C for 1 min (for *chi26* gene) or 55 °C for 1 min (for *bar* gene), 72 °C for 2 min (38 cycles); 72 °C for 8 min (1 cycle); 4 °C (overnight). The amplified products were resolved on an agarose gel (1.2%). Quick-Load 100 bp DNA ladder (New England Biolabs) was used as a DNA standard. Electrophoresis was performed at 80 V and DNA bands were visualized on a UV-transilluminator and documented by a digital camera.

### Southern blot hybridization

Southern hybridization was performed as described by Sambrook et al. [22] using ^32^P random labeling method for the four selected transgenic lines (CHI 7, CHI 14, CHI 47, and CHI 71) at T0 generation and for the non-transgenic parent (cv. Hi-Line). The genomic DNA of each line (30 µg) was digested with *Nco*I so that a fragment of the plasmid with 934 bp, a high-molecular-weight (HMW) fragment including a portion of the plasmid, and a portion of the genomic DNA with unknown sizes based on the location of the insertion (Fig. 1) could be obtained. After this, the digested DNA preparations of different transgenic lines were analyzed by 0.8% agarose gel electrophoresis. The blot was probed with the *Bam*HI fragment of pBarley/chi/bar containing the maize *ubi* promoter and *ubi* intron1 (~2 kb) (Fig. 1).

### qPCR

In order to detect *chi26* gene copy number in different transgenic lines, qPCR was carried on the leaf samples of 6-week-old T0 plantlets. The following standard equation was used to determine the number of *chi26* transgene copies in wheat haploid genome per microliter:

$${\text{No}} .\,{\text{copies}}\,({\text{genomes}}/\upmu{\text{L}}) = \frac{{{\text{DNA}}\,{\text{concentration}}\,({\text{g}}/{\text{L}})}}{{{\text{Molecular}}\,{\text{weight}}\,({\text{g}}/{\text{mol}})}} \times {\text{Avogadro's}}\,{\text{no.}} \times 10^{ - 6}$$

The genomic DNA samples were serially diluted to obtain the final quantity as 186, 93 and 18.6 ng, which were equal to 10,000, 5000 and 1000 haploid genomes/μL, respectively. qPCR was performed using 1000 copy number (18.6 ng) to be considered in the standard curve. The same quantification equation was used to generate standard curves for the absolute quantification of *chi26* transgene. The pBarley/chi/bar DNA was serially diluted to obtain the final quantity as 8.6, 0.86, 0.086 and 0.0086 ng, which were equal to 100,000, 10,000, 1000 and 100 copies/μL, respectively. All qPCRs were performed in triplets by using the gene-specific primers. The final reaction volume was 20 µL containing 1 µL DNA, 10 µL of 2× Fast SYBR Green Master Mix (Life technologies, USA), and 1 µL of 500 nM of each primer. The reactions were carried out on an Mx3005P qPCR System (Stratagene), employing following conditions: 10 min at 95 °C, 40 cycles of 30 s at 95 °C, 60 s at 60 °C, 20 s at 72 °C and 10 min at 72 °C. The optimal threshold lines were automatically determined by Mx3005p software (Agilent Technologies, Santa Clara, CA). The efficiencies of qPCR for each gene were determined by the following equation (E = (10^−1/m^ − 1) × 100) according to Radonić et al. [23], where E is the PCR efficiency; m is the slope of the log-transformed DNA quantities versus Ct values according to the equation (y (Ct) = m log(x) + b (y-intercept)). Standard deviation (SD) and coefficient of variance (CV) were estimated in MS Excel.

### Chitinase enzyme assay

Chitinase enzyme assay was performed to compare the enzyme activities in different T0 transgenic lines with that of non-transgenic parental line (cv. Hi-Line) that was recovered from tissue culture with growth conditions similar to those used to generate different transgenic lines. Total proteins were extracted from leaf tissues (0.5 g), and protein concentrations were measured according to Bradford method [24]. The chitinase enzyme assay is based on a colorimetric method [25] where colloidal chitin is used as a substrate. The substrate was prepared from crab shell chitin (Sigma) [26] and *N*-acetylglucosamine (GlcNAc) was used as the standard. The enzyme activity is defined as µmol GlcNAc min^−1^ mg^−1^ protein at 37 °C. Standard error (SE) was determined of three replicates using MS Excel.

### qRT-PCR

Total RNA was extracted from leaf tissues of selected transgenic lines at T0 and their families using TRIZOL reagent (Invitrogen, cat. 15596–018). The extracted RNA samples were treated with DNase IRQ1 RNase-free DNase (Promega, cat. no. M6101) to remove genomic DNA contamination. The expression of the integrated transgene was tested from the extracted RNA in the T0 transgenic lines that showed positive results of Basta™ resistance and expected PCR outcome. The expression of *chi26* gene was also tested for transgenic lines for T3–T9 generations. For each sample, 2 µg of total RNA was used to synthesize first strand cDNA with oligo(dT) using Revert Aid Premium Reverse Transcriptase (Thermo Scientific™ cat. no. EP0451), and *chi26* gene-specific primers (designed by GenScript Real-time PCR Primer Design, www.genscript.com/ssl-bin/app/primer) (Additional file 2: Table S2). The templates were normalized to amplify a 196-bp fragment using *actin* gene primers (Additional file 2: Table S2) for the wheat *actin* (AY663392) gene, which was used as the internal reference qRT-PCR was done in a total volume of 25-µL, which contained 1 µL cDNA, 12.5 µL 2× BIO-RAD iQTMSYBR®GreenSupermix, 0.75 μL ROX reference dye (1:500 diluted), and 1 µL 500 nM of each primer. All reactions were performed in triplicate. An Mx3005P qPCR System (Stratagene) was used with following reaction conditions: 5 min at 95 °C, 40 cycles of 30 s at 95 °C, 60 s at 60 °C, 20 s at 72 °C and 10 min at 72 °C. PCR products were examined by melt curve analysis. The *actin* expression level was quantified in each of the transgenic events and families as well as in the non-transgenic parents as the reference house-keeping gene. The amplicons obtained from the *actin* gene attained saturation between 18 and 22 cycles. The expression level of *chi26* gene relative to the *actin* gene was calculated using MxPro QPCR Software v4.10 package, which compares reaction takeoff points (cycle number). Relative mRNA abundance in each transgenic line was compared to the non-transgenic line (cv. Hi-Line) using the comparative C_t_ (2^−ΔΔCt^) method [27] with following equation: relative fold change in gene expression = 2^−ΔΔCt^, where ΔΔC_t_ = ΔC_t transgenic_ − ΔC_tnon-transgenic_, and ΔCt = C_t target gene_ − C_t reference gene_.

Data were statistically analyzed using the analysis of variance (ANOVA) via MSTATC program and multiple comparisons were made following Duncan’s Multiple Range test [28].

### Field evaluation

Five field experiments were conducted over five growing seasons (2009–2010, 2010–2011, 2011–2012, 2013–2014, and 2014–2015) to evaluate the transgenic wheat families at T4, T5, T6, T8 and T9 generations, respectively. These transgenic families harbor the barley *chi26* gene conferring resistance against wheat rusts, i.e., leaf rust (*Puccinia recondita*), stem rust (*Puccinia graminis*), yellow rust (*Puccinia striiformis*) and powdery mildew (*Blumeria graminis*). The experimental field (Gemmiza Research Station, Gharbia Governorate, Egypt, Latitude 30°47′21.98″, Longitude 31°7′33.97″) was located at a place with suitable environmental conditions in terms of temperature and humidity for the infection of the fungi. The field was surrounded by a border of a mixture of highly susceptible genotypes to wheat rusts and powdery mildew, i.e., Giza 160, Morocco and *Triticum spelta*. At booting stage, the transgenic plants were artificially exposed to an inoculum containing the mixture of the highest virulent isolates of powdery mildew, leaf, yellow, and stem rust pathotypes [29] to ensure early epidemic by rusts. The disease severity of leaf rust was scored according to the scale described by Peterson et al. [30] (where 0 = no infection, R = resistant, Tr = traces, MR = moderately resistant, MS = moderately susceptible and S = susceptible) with different intensities (5–100). In a similar vein, the disease severity of powdery mildew was recorded according to the scale described by Leath and Heun [31] (where 0 = immune, no visible signs or symptoms; 1 = highly resistant, small flecks only; 2 = resistant, chlorotic flecks evident; 3 = resistant, large flecks with chlorosis and necrosis; 4 = intermediate, mycelium and conidia barely detectable; 5 = moderately susceptible, small to moderate-sized pustules and conidia present; 6 = moderately susceptible, predominance of moderate-sized pustules and conidia present; 7 = susceptible, at least 50% of the leaf covered with large pustules and conidia; 8 = susceptible, 75–80% of the leaf covered with large pustules and conidia; and 9 = susceptible, 100% of the leaf segment covered with large pustules and conidia). A randomized complete block design with three replicates was applied for all the experiments. However, the experiments were different on the grounds of the availability of plant materials and the selection for the stable and more resistant transgenics. During the cultivation season of 2009–2010, twenty seeds each of 33 transgenic T4 families as well as the non-transgenic parental line (cv. Hi-Line) were planted in rows (40 cm apart), 1 m each. During the cultivation season of 2010–2011, transgenic families as well as the non-transgenic cv. Hi-Line were sown in plots (1.2 × 3.0 m) consisting of six rows. The seeds of selected single plants were sown in each plot in five replicates. The seeds of transgenic wheat families of subsequent generations during the following seasons were sown in plots (20 m × 2.4 m) as a demonstration experiment in five replicates. The seeds were sown at the rate of 123 kg/Ha as adopted by the National Wheat program in Egypt and all the standard agricultural practices were properly followed.

### *In silico* transgene toxicity and computational analyses

The protein sequences of the inserted genes) i.e., *chi26* and *bar*) in transgenic family CHI 14/6 were aligned to the toxicity database (Toxin and Toxin Target Database, T3DB, www.t3db.org) and to the structural database of allergenic proteins (SDAP, http://fermi.utmb.edu/cgi-bin/SDAP/sdap_14). The same transgenic family was selected for compositional analysis as compared to the non-transgenic cv. Hi-Line. Other analyses were carried out at the Regional Center for Food and Feed (RCFF), Agriculture Research Center (ARC), Giza, Egypt. Proximate compositions, such as moisture, energy, crude fiber, crude protein, crude fat, and ash content, of the investigated whole-wheat flour samples were carried out according to the AOAC (2006). The vitamin contents were determined using HPLC (Danish Official, HPLC method No. AB 189.2 (1996), National food Agency of Denmark). The official method of analysis of AOAC International no. 969.33, Chapter 41, P. 19–20, 18th ed. (2006) was used to analyze the fatty acid compositions. The amino acid contents were analyzed using the official methods of analysis of AOAC International no. 994.12, Chapter 4, pp. 17–19, 18th Edition (2005).

## Results

### Wheat transformation and recovery of transgenic families

The plant expression vector pBarley/chi/bar (Fig. 1) containing the barley *chi26* gene was transfected to the immature embryos of wheat cultivar Hi-Line by particle bombarding. Putative transgenics were screened using the herbicide Basta (1 g/L). Of the 72 transgenic lines, 14 were identified and their transgenic seeds were planted individually to obtain T1 generation. An offspring generated from a seed was considered a separate transgenic family. To facilitate the tracing of the different transgenic families across generations, nomenclature starting at the T1 generation was employed by adding new number to the original T0 identifier, e.g., CHI 14/6 refers to the T1 plant no. 6 generated from T1 seed no. 6 of the original transgenic line CHI 14. The plants obtained indifferent generations were allowed only to self pollinate.

### Gene expression and chitinase activity analyses

**Fig. 2:**
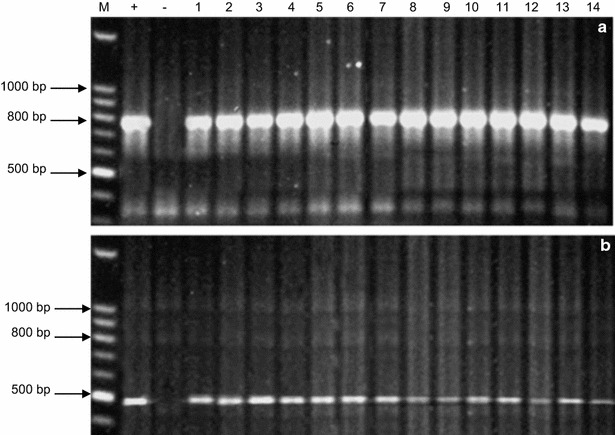
PCR products of *chi26* (800 bp) (**a**) and *bar* (400 bp) (**b**) genes from the 14 independent T0 transgenic lines (CHI 1, CHI 3, CHI 6, CHI 7, CHI 9, CHI 10, CHI 14, CHI 16, CHI 20, CHI 22, CHI 30, CHI 47, CHI 50, and CHI 71, *lanes 1–14*, respectively). M: Quick-Load^®^ 100 bp DNA Ladder (New England Biolabs), +: positive control (pBarley/chi/bar), −: negative control (non-transgenic, cv. Hi-Line)

**Fig. 3:**
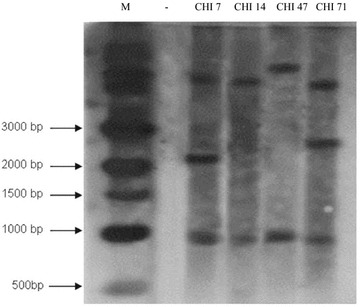
Southern blot analysis of the five independent *chi26* transgenic plants (CHI 7, CHI 14, CHI 47, and CHI 71, respectively) at T0 generation. Genomic DNA of each line was digested with *Nco*I and fragments were resolved by 0.8% agarose gel electrophoresis. The blot was probed by a *Bam*HI fragment involving a maize *ubi* promoter and maize *ubi* intron 1 (~2 kb). M; 1 Kb DNA ladder (New England Biolabs) with 0.5, 1, 1.5, 2, 3, 4, 5, 6, 8 and 10 kb, –: non-transgenic cv. Hi-Line

**Fig. 4:**
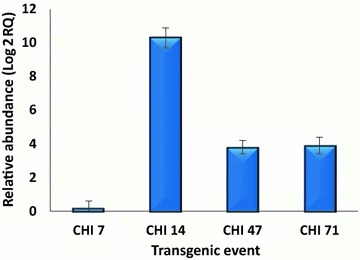
qRT-PCR analysis of relative transcript abundance of *chi26* transgene in transgenic wheat lines (CHI 7, CHI 14, CHI 47 and CHI 71) at T0 generation. Data were statistically analyzed and multiple comparisons were made following Duncan’s multiple range test [28]

**Fig. 5:**
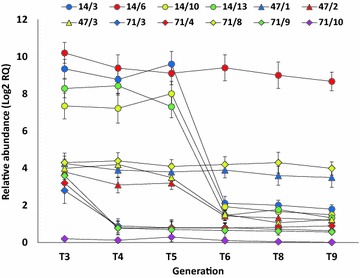
qRT-PCR analysis of relative transcript abundance of *chi26* transgene in T3–T9 transgenic wheat families (CHI 14/3, CHI 14/6, CHI 14/10, CHI 14/13, CHI 47/1, CHI 47/2, CHI 47/3, CHI 71/3, CHI 71/4, CHI 71/8, CHI 71/9 and CHI 71/10) belonging to three transgenic lines CHI 14 (*filled circles*), CHI 47 (*filled triangles*) and CHI 71 (*filled diamonds*). Data were statistically analyzed and multiple comparisons were made following Duncan’s multiple range test [28]

The PCR results (Fig. 2) confirmed the presence of the full-length *chi26* (800 bp) and *bar* (400 bp) genes in the genomic DNA of the 14 putative transgenic plants. Southern blotting was performed to confirm the integration of pBarley/chi/bar construct in the wheat genomic DNA and to detect the transgene copy number in different transgenic lines. Figure 3 shows the hybridization pattern of the generated probe with the genomic DNA of four (CHI 7, CHI 14, CHI 47, and CHI 71) out of the 14 putative transgenic lines. An expected fragment of 934 bp was detected in the genomic DNA of the transgenic lines but was completely absent in the non-transgenic line (cv. Hi-Line). Several *chi*-specific hybridizing HMW DNA fragments of variable length were observed indicating that the four transgenic lines independently originated from different embryogenic cells representing different integration events. In addition, the number of bands indicated different copy numbers of the transgene. Transgenic lines CHI 14 and CHI 47 showed the presence of a single transgene copy, while transgenic lines CHI 7 and CHI 71 indicated the occurrence of two transgene copies. The copy number was also confirmed by qPCR [32] for the *chi26* transcripts of the four transgenic lines at T0 generation. The mean C_t_ values for all sample were obtained from three replicates considering an identical threshold. The obtained values of mean C_t_, standard error (SD) and coefficient of variance (CV) are listed in Table 1. The levels of *actin* gene expression in different transgenic lines and the non-transgenic control were identical. qRT-PCR analysis at T0 revealed different patterns for the four transgenic lines. The line CHI 14 showed the maximum transcript abundance followed by the lines CHI 71 and CHI 47, while the line CHI 7 showed the lowest value (Fig. 4). qRT-PCR for the selected families of the three transgenic lines CHI 14, CHI 47 and CHI 71 (i.e., CHI 14/3, CHI 14/6, CHI 14/10; CHI 14/13, CHI 47/1, CHI 47/2, CHI 47/3, CHI 71/3, CHI 71/4, CHI 71/8, CHI 71/9 and CHI 71/10) was also carried out to detect any possibility of gene silencing in the gene families across different generations up to T9 (Fig. 5). The results indicated that *chi26* gene was silenced in all transgene families in different generations, except for CHI 14/6, CHI 47/1 and CHI 71/8. The transgene in these families was not silenced across the nine studied generations. The gene silencing in the families of the transgenic line CHI 71 took place early at T3 (i.e., CHI 71/10) and T4 (i.e., CHI 71/3, CHI 71/4 and CHI 71/9), while it took longer and occurred at T6 in the families of transgenic lines CHI 14 (i.e., CHI 14/3, CHI 14/10 and CHI 14/13) and CHI 47 (i.e., families CHI 47/2 and CHI 47/3). As shown in Table 2, the chitinase activity was higher in the T0 transgenic lines than their parental non-transgenic line (Hi-Line). There were variations in enzyme activity among the tested transgenic lines. The line CHI 14 had the highest activity (15.1 U/mg) followed by the lines CHI 47 and CHI 71 (14.3 and 12.7 unit/mg, respectively). These results concord with those of qRT-PCR for *chi26* transcript abundance (Fig. 4) in different transgenic lines.

**Table 1:** Estimated *chi26*-transgene copy number in the T0 transgenic plants determined byqRT-PCR

Line no.	(Quantity) 100 target copies	Copy average ± SD	CV of copy average	Estimated copy number
CHI 7	0.93E + 03	1.27^E+03^ ± 4.81^E+02^	0.38	2
	1.28E + 03			
	1.61E + 03			
CHI 14	0.82E + 03	0.95^E+03^ ± 1.80^E+02^	0.19	1
	0.94E + 03			
	1.08E + 03			
	1.55E + 03			
	1.43E + 03			
CHI 47	0.64E + 03	0.78^E+03^ ± 1.98^E+02^	0.25	1
	0.8E + 03			
	0.90E + 03			
CHI 71	1.43E + 03	1.45^E+03^ ± 1.56^E+02^	0.11	2
	1.35E + 03			
	1.57E + 03			
Hi-Line (non-transgenic)	–	–	–	–
	–			
	–			

*SD* standard deviation, *CV* coefficient of variance

**Table 2:** Mean chitinase activity in T0 transgenic wheat plants with barley *chi26* gene

Transgenic line	Mean chitinase activity (U/mg of protein) ± SE
CHI 7	3.7 ± 0.5
CHI 14	15.1 ± 1.6
CHI 47	14.3 ± 0.8
CHI 71	12.7 ± 1.8
Hi-Line (non-transgenic)	3.0 ± 0.75

The values are expressed as mean ± standard error (SE) of three technical replicates

### Field evaluation of rusts and powdery mildew disease resistance

**Fig. 6:**
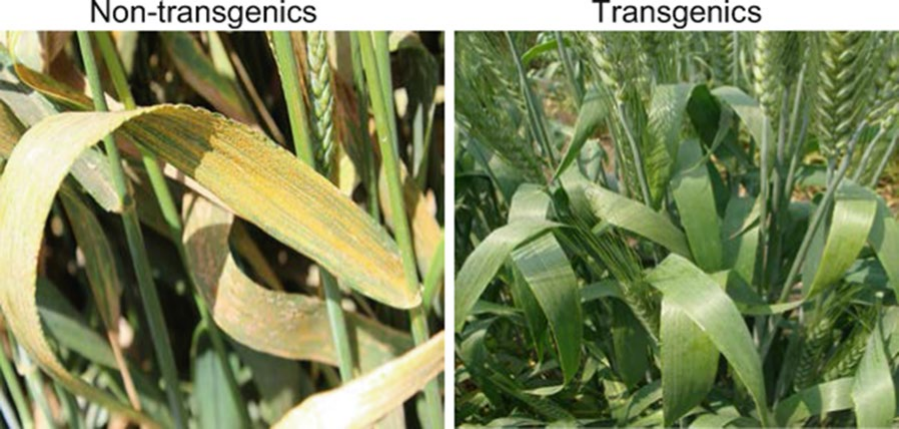
Comparison between *chi26*-transgenic (CHI 14/6) and non-transgenic wheat plants grown at the field station during the season 2014–2015 indicating the combined effects of the three fungi: powdery mildew, and leaf and yellow rusts

The resistance of the selected transgenic wheat families against two wheat rusts, leaf rust (*Puccinia recondita*) and yellow rust(*Puccinia striiformis*), and powdery mildew (*Blumeria graminis*), was evaluated in five field experiments through five growing seasons (2009–2010, 2010–2011, 2011–2012, 2013–2014 and 2014–2015) at Gemmiza Research Station, Egypt. As shown in Table 3, the degree of resistance varied among the transgenic families against the combined infection of the three fungi across different seasons. In addition, the resistance level against different fungi in a given generation varied among the families of the same transgenic event. During cultivation season of 2009–2010, 25 CHI families belonging to four independent homozygous transgenic lines (CHI 7, CHI 14, CHI 47 and CHI 71) were evaluated. In this season, the environmental conditions at Gemmiza station were not suitable for the growth of yellow rust, as there were no yellow rust symptoms on the cultivars grown on the susceptible border, but the conditions were optimal for leaf rust and powdery mildew infections. All the families of transgenic line CHI 14 were considerably resistant to leaf rust and powdery mildew infections. In contrast, all the families of line CHI 7 were susceptible to the infection of leaf rust and powdery mildew; these line, therefore, were not proceeded to field testing for further generations. Three families of line 47 (CHI 47/1, CHI 47/2, and CHI 47/3) and one family of line CHI 71 (CHI 71/8) were resistant to leaf rust and powdery mildew both (Table 3). The other families of the line CHI 47 were highly susceptible to the two fungi, so were excluded from field testing in further generations. The families of line CHI 71 ranged from being moderately resistant to highly susceptible to leaf rust and being susceptible to highly susceptible to powdery mildew, during this and the following four seasons. The highly susceptible family CHI 71/10 was used in field-testing for further generations, as a *chi26* non-expressing transgenic family in addition to the wild type. The data recorded during the 2010–2011 season for the selected 12 transgenic families were similar to those obtained in the first season against the two fungi. The resistance levels against yellow rust were parallel to those were against leaf rust. In the season 2011–2012, high susceptibility against the three fungi started to arise in the five (CHI 14/3, CHI 14/10, CHI 14/13, CHI 47/2 and CHI 47/3) of the eight resistant families. The results obtained during the seasons 2013–2014 and 2014–2015 for the 12 families were almost similar to those obtained during 2011–2012 for the selected 12 families. The results for the season 2009–2010 indicated that selective transgene silencing occurred in earlier generations for the family lines, CHI 7 and CHI 71, but excepting CHI 71/8. The results obtained during the season 2014–2015 and two following growing seasons indicated possible occurrence of gene silencing in the families of the lines CHI 14 and CHI 47, except CHI 14/6 and CHI 47/1. The performance of *chi26*-expressing transgenic line (e.g., CHI 14/6) versus non-transgenic line under combined infection is shown in Fig. 6. This observation is also supported by a video captured at the field station during the last season to indicate the performance of the co-existing transgenic and non-transgenic plants during the season 2014–2015 (Additional file 3: Movie 1). Regarding stem rust (*Puccinia graminis*), the parental cultivar Hi-Line was natively resistant to the pathotypes available at the research station. Therefore, the effect of *chi26* gene towards stem rust resistance could not be evaluated through the field experiments carried out. The data presented in Table 3 for the field experiments concurred well with those obtained by qRT-PCR, in terms of gene expression levels of different transgenic families across generations (Fig. 5). As a result, the transgenic family CHI 14/6 was selected for further testing of possible transgene toxicity and substantial equivalence.

**Table 3:** Evaluation of field data for T4, T5, T6, T8, and T9 transgenic wheat families as well as their parental non-transgenic cv. Hi-Line, for resistance against wheat rusts, leaf rust (*Puccinia triticina*) and yellow rust (*Puccinia striiformis f*. sp. tritici), and powdery mildew (*Blumeria graminis*) under artificial inoculation at the field of Gemmiza Research Station during the growing seasons 2009–2010, 2010–2011, 2011–2012, 2013–2014 and 2014–2015

Growing season		2009–2010			2010–2011			2011–2012			2013–2014			2014–2015		
Generation	Family no.	(T4)			(T5)			(T6)			(T8)			(T9)		
Transgenic line no.		Fungus														
		Lr	Yr	Pm	Lr	Yr	Pm	Lr	Yr	Pm	Lr	Yr	Pm	Lr	Yr	Pm
7	7/4	80S	NA	8	–	–	–	–	–	–	–	–	–	–	–	–
	7/7	80S	NA	8	–	–	–	–	–	–	–	–	–	–	–	–
	7/12	80S	NA	8	–	–	–	–	–	–	–	–	–	–	–	–
	7/30	80S	NA	8	–	–	–	–	–	–	–	–	–	–	–	–
	7/33	80S	NA	8	–	–	–	–	–	–	–	–	–	–	–	–
14	14/3	R	NA	0	R/0	0	0	5MR	MR/Tr	2	20MR	5MR	2	20MR	5MR	2
	14/6	0	NA	0	0	0	0	0	0	0	0	0	0	0	0	0
	14/10	R/0	NA	0	R/0	0	0	30MR	30MR	3	MS/MR	20MS	3	5MS	5MS	3
	14/13	0	NA	0	R	R	0	30MR	30MR	3	30MR	20MR	3	30MR	5MR	3
47	47/1	R	NA	0	R	R/0	0	R	Tr/R	0	R	Tr/R	0	R/0	R	0
	47/2	Tr/R	NA	0	Tr	Tr/R	0	20MS	MS/MR	3	30MS	MS/MR	3	20MS	20MS	3
	47/3	R	NA	0	Tr/R	Tr	0	MS/MR	20MS	3	20MS	20MS	3	30MS	5S	3
	47/5	60S	NA	8	–	–	–	–	–	–	–	–	–	–	–	–
	47/6	60S	NA	8	–	–	–	–	–	–	–	–	–	–	–	–
	47/7	80S	NA	8	–	–	–	–	–	–	–	–	–	–	–	–
	47/8	60S	NA	8	–	–	–	–	–	–	–	–	–	–	–	–
	47/9	60S	NA	8	–	–	–	–	–	–	–	–	–	–	–	–
	47/10	80S	NA	8	–	–	–	–	–	–	–	–	–	–	–	–
	47/11	60S	NA	8	–	–	–	–	–	–	–	–	–	–	–	–
	47/12	60S	NA	8	–	–	–	–	–	–	–	–	–	–	–	–
71	71/3	MS/MR	NA	6	MS	5S	6	60S	5S	8	60S	20S	8	60S	20S	8
	71/4	MS	NA	6	20S	20MR	6	60S	30MS	6	60S	30MS	6	60S	30S	8
	71/8	R	NA	0	R	Tr	0	R/0	Tr/R	0	Tr/R	R	0	R	R/0	0
	71/9	30S	NA	6	60S	MS/MR	6	80S	MS	6	80S	5S	8	80-S	20S	8
	71/10	80S	NA	8	80S	60S	8	80S	80S	8	80S	80S	8	80S	80S	8
Hi-Line		80S	NA	9	80S	80S	9	80-S	80S	9	80-S	80S	9	80-S	80S	9

*Lr* leaf rust, *Yr* yellow rust, *Pm* powdery mildew. 0 (Lr and Yr) = no infection, R = resistant, Tr = traces, MR = moderate resistant, MS = moderate susceptible and S = susceptible. NA = not applicable. 0 (Pm) = immune, no visible signs or symptoms; 1 = highly resistant, smallflecks only; 2 = resistant, chlorotic flecks evident; 3 = resistant, large flecks with chlorosis and necrosis; 4 = intermediate, mycelium and conidia barely detectable; 5 = moderately susceptible, small to moderate-sized pustules and conidia present; 6 = moderately susceptible, predominance of moderate-sized pustules and conidia present; 7 = susceptible, at least 50% of the leaf covered with large pustules and conidia; 8 = susceptible, 75–80% of the leaf covered with large pustules and conidia; and 9 = susceptible

### *In silico* analysis of possible transgene toxicity and substantial equivalence testing

The protein sequences of the inserted genes (*chi* and *bar*) were aligned to the toxicity (Toxin and Toxin Target Database, T3DB, www.t3db.org) and allergenicity (SDAP, http://fermi.utmb.edu/cgi-bin/SDAP/sdap_14) databases, and no homology was observed between the proteins encoded by both the genes and any known toxic compound. The deduced amino acid sequence of the protein encoded by *bar* gene showed no homology with any known allergen. However, there was some homology (~35%) between the deduced amino acid sequence of the protein encoded by *chi26* gene and an allergen protein in banana. Phenotypic changes, in terms of the performance of the transgenic plants, were observed across the different seasons in comparison to their parental non-transgenic plants following the guidelines of the European Food Safety Authority (EFSA) for genetically modified organisms. The results indicated that there were no observable phenotypic differences within transgenics and between the transgenics and non-transgenic parental plants. The overall mode of reproduction and the survivability rate of the transgenic plants were the same as to the non-transgenics. The proximate analysis and compositions of vitamins, fatty acids, and amino acids of CHI 14/6 transgenic family were similar to those of the parental Hi-Line (Table 4). The *chi26*-transgenic family showed a slight increase in protein and fat contents compared to the wild type, and slightly lower levels of vitamins B1 and B2 and fatty acids, except linolenic acid. The amino acid profiles were not affected by the presence of the transgene.

**Table 4:** Comparison between proximate analysis, and vitamin, fatty acid and amino acid contents of CHI 14/6 and non-transgenic cv. Hi-Line

	Hi-Line	CHI14/6		Hi-Line	CHI14/6
Proximate analysis (%)			Vitamins (mg/kg)		
Moisture	10.40*	10.40	Vit B1	5.100^A^	5.000^A^
Ash	1.37	1.72	Vit B2 (mg/kg)	0.539^A^	0.480^A^
Crude fiber	2.71	2.6	Para-aminobenzoic acid	770.000^A^	800.000^A^
Crude protein	15.4	15.6			
Crude fat	2.16	2.25			
Fatty acids (%)			Amino acids (%)		
Caprylic acid	0.2	–	Alanine	0.51^A^	0.56^A^
Capric acid	0.2	–	Arginine	0.77^A^	0.72^A^
Myristic acid	0.1	–	Aspartic acid	0.83^A^	0.76^A^
Palmitic acid	21.7	17.2	Glutamic acid	4.15^A^	4.25^A^
Palmitoleic acid	0.6	–	Glycine	0.63^A^	0.73^A^
Stearic acid	2.9	2	Histidine	0.34^A^	0.33^A^
Oleic acid	17.6	16.2	Isoleucine	0.49^A^	0.45^A^
Vaccenic acid	1.2	1	Leucine	0.96^A^	0.91^A^
Linoleic acid	51	60.7	Lysine	0.39^A^	0.38^A^
Linolenic acid	3.2	2.9	Phenylalanine	0.66^A^	0.66^A^
Gadoleic acid	0.6	0.7	Proline	3.73^A^	1.33^A^
Arachidic acid	–	0.1	Serine	0.63^A^	0.57^A^
C20:1ω 7	0.4	–	Threonine	0.42^A^	0.38^A^
Non identified fatty acid	0.3	0.2	Tyrosine	0.49^A^	0.48^A^
			Valine	0.62^A^	0.65^A^

* Data of three biological replicates were statistically analyzed and multiple comparisons were made following Duncan’s Multiple Range test [28]. Numbers in the same row followed by the same letter are not significantly different

## Discussion

In many countries, wheat serves as an important staple crop. Depending on the climatic conditions, it can be infested by a wide variety of fungi [33]. The conventional method to control fungal parasites is mainly dependent on the extensive use of chemical fungicides, which are harmful to the ecological system, leave considerable residual toxicity to humans and animals, and increase the production cost. Moreover, fungi have developed resistance to some of the available commercial fungicides [34]. Therefore, it is desirable to develop resistant cultivars by the introduction of resistance genes employing conventional breeding and genetic transformation. *Chitinase* is one of the defense genes that can abort fungal growth by degrading the chitin of growing hyphae by chitinase [35]. Several studies have demonstrated that enhanced *chitinase* levels in transgenic plants can increase the plant resistance against several fungal pathogens. The plants tested for this approach are rice [15], ryegrass [36], tomato [37], peanut [38], banana [39], finger millet [40], and melon [16]. The first attempt to produce transgenic wheat with *chitinase* gene dates back to 1998, which was unsuccessful due to the silencing and loss of *chitinase* expression in T1 and subsequent generations [41]. Bliffeld et al. [17] generated transgenic wheat plants constitutively expressing antifungal barley-seed class II chitinase. The transfected plants were able to reduce the severity of the infection by *Erysiphe graminis* in transgenic wheat plants. Another study achieved the same results [42], wherein the number of the spore-containing colonies was reduced on the leaves of barley class II chitinase transgenic wheat lines (30–50%) at an inoculum density of approximately 100 mildew (*Erysiphe graminis*) or 80 rust (*Puccinia recondita*) spores per cm^2^. The evaluation of transgenic lines overproducing chitinase and β-1,3-glucanase showed resistance to *Fusarium graminearum* in greenhouse conditions. However, the same lines failed to show any resistance under actual field conditions [43]. On the other hand, Shin et al. [19] generated seven transgenic wheat lines expressing the barley class II chitinase. These lines exhibited high Type II Fusarium head blight (FHB) resistance in the greenhouse and two of them also showed enhanced resistance even in the field.

In this study, we inserted the barley *chi26* coding cDNA into the wheat genome (cv. Hi-Line) via biolistic bombardment. The gene integration in the transgenic plants was confirmed by the Southern blotting and the gene expression was confirmed by qRT-PCR and chitinase activity assays. To the best of our knowledge, the present study is the first in evaluating chitinase-expressing transgenic wheat in natural field conditions for five seasons at a hot spot region (Gemmiza Research Station, Gharbia Governorate, Egypt) suitable for testing wheat rusts and powdery mildew. According to the field data obtained, it can be concluded that some transgenic wheat families with *chi26* gene showed high resistance to rust diseases, including leaf rust (*Puccinia recondita*), yellow rust (*Puccinia striiformis*) and powdery mildew (*Blumeria graminis*) (Table 3). There were some variations in the resistance in the field among the families of the same transgenic event in a given season (Table 3). This behavior could also be supported by the data of the transgene expression level obtained by qRT-PCR for different families across different seasons (Fig. 5). The results indicated that transcription of *chi26* was silenced in several families at T3, T4, and T6 generations. This conclusion is supported by the data generated during field-testing. Stability in transgene expression was observed at T7 up to T9. The phenomenon of gene silencing has also been reported earlier [41], where the authors found that the majority of the T1 progeny showed very limited or almost no *chitinase* gene expression despite carrying an intact transgene. They ascribed this behavior to the use of CaMV–35S promoter driving the transgene. Transgene silencing has also been reported by other investigators [44, 45], with this as well other promoters, e.g., CaMV–35S [45, 46], *Act1D* [44], and maize *ubi1* [43, 47], with different transformation methods, such as *Agrobacterium*-mediated transformation of oilseed rape [48, 49] and microprojectile bombardment for wheat [43, 47]. These reports and the current study support the conclusion that transgene silencing is not correlated to any specific promoter or transformation method used. The high frequency of gene silencing might occur via homology-dependent silencing [50], tandem insertions, or genomic DNA could be interspersed between transgenic sequences [51–53]. Another explanation is the occurrence of promoter methylation, where expression level can be largely linked to the degree of promoter methylation [43, 47]. The results of the current study are in agreement with the latter possibility because we found fluctuations in *chi26* expression in subsequent generations for families generated from the same transgenic event. In addition, we observed no direct correlation between the transgene copy number and the silencing level, where CHI 14 and CHI 47 were found to have one transgene copy, while CHI 71 was having two. Similarly, Rooke et al. [54] found no correlation between the expression of the HMW glutenin subunit gene and its copy number. Despite the occurrence of transgene silencing, there was an overall stability in the degree of resistance in a given transgenic event across all tested generations (Table 3). If a given transgenic line could escape silencing up to T6 generation, then it is highly possible that the subsequent generations will exhibit stable expression levels. Transgene silencing in wheat is a random process and has no correlation with promoter, transgene copy number, or even position effect [43]. However, further investigation is indispensable to understand the mechanism of transgene silencing. In accordance to the possible correlation between chitinase activity and fungal disease resistance, several studies have claimed that transgenic plants with higher levels of *chitinase* transcript accumulation show higher levels of disease resistance and chitinase activity [36]. On the other hand, some studies carried out in rice, tobacco, and cotton, have reported that the level of chitinase activity does not always correlate with the degree of fungal resistance [55, 56]. More recently, a simple method namely WAC, based upon the specific binding of the plant lectin wheat germ agglutinin to fungal chitin, was used to detect disease resistance against *Puccinia graminis* f. sp. *Tritici* [57]. World Health Organization (WHO), Food and Agriculture Organization (FAO), and Organization for Economic Cooperation and Development (OECD) follow the principle of the “substantial equivalence” for genetically modified food safety assessment [49]. In the current study, a comparative analysis between *chi26* transgenic line and its parental non-transgenic genotype (cv. Hi-Line) was performed. None of the transgenic plants exhibited any morphological change as compared to the non-transgenic parental genotype. They were self-fertile and had normal seed setting. In addition, no significant differences in agronomic traits between the transgenic and non-transgenic families were observed under normal conditions (Additional file 4: Table S3). One of the transgenic families (CHI 14/6) showed stable resistance across the studied generations, and consequently, was chosen for subsequent proximate and nutritional analyses(Additional file 5: Figure S1). Moisture content is one of the most important considerations while evaluating the quality of wheat for long terms to rage and milling quality of the grain [48]. The moisture content in the commercial lots of wheat ranges between 8 and 18%, depending on the prevalent weather at the time of harvest [57, 58]. The moisture content of CHI14/6 and Hi-Line was the same (Table 4). In addition, the ash, protein, and fat percentages were also similar. The slight variation in fatty acid and amino acid profiles is within naturally occurring variability range and thus acceptable [49].

## Conclusion

Our results indicate that the resistance against wheat rusts and powdery mildew can be achieved via transgenesis with the use of the barley *chi26* gene. The recovered transgenic family CHI 14/6 can be utilized in breeding programs towards the production of wheat cultivars resistant to rusts and powdery mildew.

## Additional files

**Additional file 1: Table S1.** Specific primers to amplify *chi26* and *bar* genes.

**Additional file 2: Table S2.** Specific primers to amplify *chi26* and *actin* genes for qPCR.

**Additional file 3: Movie 1.** The performances of *chi26* transgenics versus non-transgenics under combined infection with rusts and powdery mildew.

**Additional file 4: Table S3.** Means of yield related traits of the T4 transgenic families as well as their parental non-transgenic genotype (cv Hi-Line) under field conditions in cultivated seasons 2009/2010.

**Additional file 5: Fig. S1.** Whole figure for Genomic Southern blot analysis of the five independent *chi26* transgenic plants CHI 7, CHI 30, CHI 14, CHI 47 and CHI 71, respectively) at the T0 generation. Genomic DNA of each line was digested with *Nco*I and fragmented by 0.8% agarose gel electrophoresis. The blot was probed with a *Bam*HI fragment involving a maize *ubi* promoter and maize *ubi* intron 1 (~2 kb). M; 1 Kb DNA ladder (New England Biolabs) with 0.5, 1, 1.5, 2, 3, 4, 5, 6, 8 and 10 kb, –; non-transgenic cv. Hi-Line.

## Abbreviations

G168: Giza 168
ELISA: enzyme linked-immunosorbent assay
