# Supplementary material for: Developing transgenic wheat to encounter rusts and powdery mildew by overexpressing barley chi26 gene for fungal resistance
Source: Plant Methods. 2017 May 22;13:41. doi: 10.1186/s13007-017-0191-5 (PMC5441082; doi:10.1186/s13007-017-0191-5)
Supplement: Supplementary file 1 — Additional file 1: Table S1. Specific primers to amplify chi26 and bar genes. [file 13007_2017_191_MOESM1_ESM.docx]

Table S1. Specific primers to amplify *chi26* and *bar* genes.

No. Primer name Primer sequence

1 *Chi26* (forward) 5`TAT TAT CAT ATG AGA TCG CTC GCG GTG GTGGTG 3׳

2 *Chi26* (reverse) 5`TAT ATA CAT ATG GGA TCC ATA GGC GAA GGG TCT 3׳

3 *bar* (forward) 5^׳^ ACC ATC GTC AAC CAC TAC AT 3^׳^

4 *bar* (reverse) 5׳ CAG ATC TCG GTG ACG GG 3׳
