# Supplementary material for: Developing transgenic wheat to encounter rusts and powdery mildew by overexpressing barley chi26 gene for fungal resistance
Source: Plant Methods. 2017 May 22;13:41. doi: 10.1186/s13007-017-0191-5 (PMC5441082; doi:10.1186/s13007-017-0191-5)
Supplement: Supplementary file 2 — Additional file 2: Table S2. Specific primers to amplify chi26 and actin genes for qPCR. [file 13007_2017_191_MOESM2_ESM.docx]

Table S2. Specific primers to amplify *chi26* and *actin* genes for qPCR.

No. Primer name Primer sequence

1 *Chi26* (forward) 5`-TAG CAC AGA CCT CCC ACG A-3`

2 *Chi26* (reverse) 5`-CCG TCA TCC AGA ACC AGA-3`

3 *a*ctin (forward) 5`-TGA CGT GGA TAT CAG GAA GG-3`

4 *actin* (reverse) 5`-GCT GAG TGA GGC TAG GAT GG-3`
