# Supplementary material for: Developing transgenic wheat to encounter rusts and powdery mildew by overexpressing barley chi26 gene for fungal resistance
Source: Plant Methods. 2017 May 22;13:41. doi: 10.1186/s13007-017-0191-5 (PMC5441082; doi:10.1186/s13007-017-0191-5)
Supplement: Supplementary file 4 — Additional file 4: Table S3. Means of yield related traits of the T4 transgenic families as well as their parental non-transgenic genotype (cv Hi-Line) under field conditions in cultivated seasons 2009/2010. [file 13007_2017_191_MOESM4_ESM.docx]

Table S3. Means of yield related traits of the T4 transgenic families as well as their parental non-transgenic genotype (cv Hi-Line) under field conditions in cultivated seasons 2009/2010.

Transgenic Transgenic 2009/201

Line no. family no. ____________________________________________________________

PH SW NT/P GW/P 1000 GW

7 7/4 114.2^A-J^ 3.015^A^ 12.40^AB^ 20.710^A^ 33.17^NO^

7/7 116.4^A-F^ 2.501^A-M^ 11.40^AB^ 12.690^C-I^ 35.71^J-M^

7/12 114.6^A-J^ 2.967^A^ 10.00^A-E^ 14.600^A-H^ 37.98^F-I^

7/30 113.6^B-K^ 2.698^A-C^ 6.80^DE^ 9.543^F-J^ 44.24^B^

7/33 111.0^C-K^ 2.473^A-G^ 9.40^A-E^ 12.920^C-I^ 47.43^A^

14 14/3 110.8^D-K^ 2.985^A^ 13.40^A^ 20.720^A^ 37.09^H-L^

14/6 104.4^L^ 2.629^A-D^ 13.20^A^ 12.980^C-I^ 37.41^G-J^

14/10 110.6^E-K^ 1.940^E-K^ 13.20^A^ 17.500^A-D^ 35.56^J-M^

14/11 111.6^C-K^ 2.250^C-I^ 12.60^AB^ 15.620^A-G^ 32.91^NO^

14/13 113.8^B-K^ 2.450^A-H^ 9.60^A-E^ 10.780^D-J^ 36.82^H-L^

47 47/1 109.2^H-L^ 2.268^C-I^ 9.40^A-E^ 9.949^F-J^ 37.47^G-J^

47/2 115.4^A-H^ 1.910^E-K^ 11.80^ABC^ 12.280^C-I^ 37.21^H-K^

47/3 113.4^B-K^ 2.065^D-J^ 12.60^AB^ 15.070^A-G^ 38.26^F-I^

47/4 110.5^E-K^ 1.851^H-K^ 10.50^A-D^ 13.450^B-I^ 39.36^D-G^

47/5 112.6^C-K^ 1.349^KL^ 9.60^A-E^ 11.480^D-J^ 41.35^C^

47/6 109.0^I-L^ 2.513^A-E^ 10.00^A-E^ 12.700^C-I^ 38.84^E-H^

47/7 110.2^F-K^ 1.853^H-K^ 11.25^ABC^ 7.119^JI^ 39.68^C-F^

47/8 111.4^C-K^ 2.059^D-J^ 9.80^A-E^ 12.050^D-I^ 32.64^O^

47/9 116.6^A-E^ 1.507^JKL^ 9.80^A-E^ 12.770^C-I^ 38.47^F-I^

47/10 116.0^A-G^ 1.880^G-K^ 11.60^ABC^ 16.070^A-F^ 41.01^CD^

47/11 111.0^C-K^ 2.328^C-M^ 6.00^E^ 4.991^J^ 37.44^G-J^

47/12 111.6^C-M^ 2.376^B-I^ 11.20^ABC^ 18.900^ABC^ 44.44^B^

71 71/1 115.8^A-G^ 2.388^B-I^ 6.40^DE^ 8.862^G-J^ 36.81^H-L^

71/2 112.2^C-K^ 1.910^F-K^ 12.60^AB^ 11.530^D-J^ 37.01^H-L^

71/3 120.0^A^ 3.018^A^ 12.00^ABC^ 16.950^A-E^ 40.57^CDE^

71/4 118.8^AB^ 1.901^F-K^ 10.40^A-D^ 10.980^D-J^ 36.44^I-M^

71/5 111.6^C-K^ 1.612^JKL^ 10.00^A-E^ 9.504^F-J^ 34.57^MN^

71/6 107.6^KL^ 1.919^E-K^ 13.00^AB^ 7.161^IJ^ 35.78^J-M^

71/7 110.8^D-K^ 1.573^JKL^ 8.00^CDE^ 8.082^HIJ^ 35.22^KLM^

71/8 110.0^G-L^ 1.953^E-J^ 8.60^B-E^ 7.632^IJ^ 34.45^MNO^

71/9 117.0^A-D^ 2.089^D-J^ 9.20^A-E^ 14.720^A-H^ 37.57^G-J^

71/10 114.2^A-J^ 2.361^B-I^ 12.80^AB^ 15.540^A-G^ 35.10^LM^

Hi-Line 115.2^A-I^ 2.928^AB^ 13.20^A^ 19.780^AB^ 39.40^D-G^

Means within column (for each trait), followed by the same letter(s) are not significantly different by Duncan’s New Multiple Range Test (P<0.05). PH = Plant height (cm), SW; main spike weight (g), NT/P = number of tillers/plant, GW/P = grain weight/plant (g), 1000 GW = 1000 grain weight (g).

The data was statistically analyzed using the analysis of variance (ANOVA) via MSTATC program. The differences between means were compared using Duncan multiple test [28].
