# Supplementary material for: Developing transgenic wheat to encounter rusts and powdery mildew by overexpressing barley chi26 gene for fungal resistance
Source: Plant Methods. 2017 May 22;13:41. doi: 10.1186/s13007-017-0191-5 (PMC5441082; doi:10.1186/s13007-017-0191-5)
Supplement: Supplementary file 5 — Additional file 5: Fig. S1. Whole figure for Genomic Southern blot analysis of the five independent chi26 transgenic plants CHI 7, CHI 30, CHI 14, CHI 47 and CHI 71, respectively) at the T0 generation. Genomic DNA of each line was digested with NcoI and fragmented by 0.8% agarose gel electrophoresis. The blot was probed with a BamHI fragment involving a maize ubi promoter and maize ubi intron 1 (~2 kb). M; 1 Kb DNA ladder (New England Biolabs) with 0.5, 1, 1.5, 2, 3, 4, 5, 6, 8 and 10 kb, –; non-transgenic cv. Hi-Line. [file 13007_2017_191_MOESM5_ESM.docx]

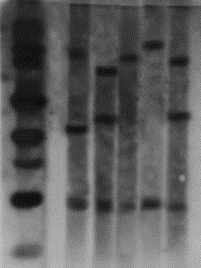


M - CHI 7 CHI 30 CHI14 CHI 47 CHI 72

1000 bp

500bp

1500 bp

2000 bp

3000 bp

Fig. S1. Whole figure for Genomic Southern blot analysis of the five independent *chi26* transgenic plants CHI 7, CHI 30, CHI 14, CHI 47 and CHI 71, respectively) at the T0 generation. Genomic DNA of each line was digested with *NcoI* and fragmented by 0.8% agarose gel electrophoresis. The blot was probed with a *BamHI* fragment involving a maize *ubi* promoter and maize *ubi* intron 1 (~ 2 kb). M; 1 Kb DNA ladder (New England Biolabs) with 0.5, 1, 1.5, 2, 3, 4, 5, 6, 8& 10 kb, -; non-transgenic cv. Hi-Line.
